# The position of the longest intron is related to biological functions in some human genes

**DOI:** 10.3389/fgene.2022.1085139

**Published:** 2023-01-10

**Authors:** Pavel Dvorak, Vojtech Hanicinec, Pavel Soucek

**Affiliations:** ^1^ Department of Biology, Faculty of Medicine in Pilsen, Charles University, Pilsen, Czechia; ^2^ Biomedical Center, Faculty of Medicine in Pilsen, Charles University, Pilsen, Czechia; ^3^ Institute of Medical Genetics, University Hospital Pilsen, Pilsen, Czechia; ^4^ Toxicogenomics Unit, National Institute of Public Health, Prague, Czechia

**Keywords:** human genome, gene structure, introns, longest intron, gene function, gene expression

## Abstract

The evidence that introns can influence different levels of transfer of genetic information between DNA and the final product is increasing. Longer first introns were found to be a general property of eukaryotic gene structure and shown to contain a higher fraction of conserved sequence and different functional elements. Our work brings more precise information about the position of the longest introns in human protein-coding genes and possible connection with biological function and gene expression. According to our results, the position of the longest intron can be localized to the first third of introns in 64%, the second third in 19%, and the third in 17%, with notable peaks at the middle and last introns of approximately 5% and 6%, respectively. The median lengths of the longest introns decrease with increasing distance from the start of the gene from approximately 15,000 to 5,000 bp. We have shown that the position of the longest intron is in some cases linked to the biological function of the given gene. For example, DNA repair genes have the longest intron more often in the second or third. In the distribution of gene expression according to the position of the longest intron, tissue-specific profiles can be traced with the highest expression usually at the absolute positions of intron 1 and 2. In this work, we present arguments supporting the hypothesis that the position of the longest intron in a gene is another biological factor modulating the transmission of genetic information. The position of the longest intron is related to biological functions in some human genes.

## Introduction

The exon-intron architecture of genes constitutes one of the basic characteristics of the eukaryotic domain of life. While the function of exons as regions coding for the consequence of amino acids in polypeptide strings is defined clearly, the importance of introns has been a matter of research for many years and the repertoire of their functions has been unraveled gradually. We now have evidence that introns can influence different levels of transfer of genetic information between DNA and the final product (Chorev and Carmel, 2012). In the case of proteins, this may be mediated, for example, by the presence of transcription enhancers and silencers or by the still not fully elucidated mechanism of intron-mediated enhancement (IME) (Nott et al., 2003; Hube and Francastel, 2015; Zalabak and Ikeda, 2020).

Longer first introns were found to be a general property of eukaryotic gene structure (Bradnam and Korf, 2008; Zhu et al., 2009). Significantly longer introns were detected in 5′ untranslated regions of genes in comparison with coding regions and 3′ untranslated regions, and a sharp drop in intron size at the boundary between 5′ untranslated regions and coding regions was reported (Chung et al., 2006; Hong et al., 2006). The long first introns were shown to contain a higher fraction of conserved sequence and different functional elements such as CpG islands, chromatin marks (e.g., H3K4me1) (Jo and Choi, 2019) and transcription key motifs (e.g., TATA box) (Li et al., 2012) in vertebrates and several other species including *Drosophila* and *Arabidopsis* (Marais et al., 2005; Gaffney and Keightley, 2006; Vinogradov, 2006; Rose et al., 2008; Park et al., 2014; Rose, 2019).

Our work develops this area of research in a further direction. The main questions we asked at the outset that were not answered in the literature included the following. In what percentage of human genes is the first intron the longest of all introns and in what percentage are the longest introns at additional positions? What is the distribution of absolute *versus* relative positions of the longest introns in human genes? What are the median lengths of the longest introns depending on the different position in the gene? Is the absolute or relative position of the longest intron related to the biological function or expression of the given gene? We addressed the answers to these questions in the current article.

## Materials and methods

### A set of studied human genes

The list of all human gene families and their members was downloaded from the HGNC database (HUGO Gene Nomenclature Committee; https://www.genenames.org/; accessed 1/10/2020). One gene was selected from each protein-coding gene family. This was usually the gene placed first in the list. In case the same gene was listed first in multiple gene families, one of the following representatives was selected. The resulting gene selection (Study dataset) contained 1,427 unique genes representing all human protein-coding families. The lengths of all exons and introns of the major isoforms, with transcript Flags MANE Select v0.95 and Ensembl Canonical, of these selected genes were obtained from the Ensembl database (https://www.ensembl.org/index.html; Ensembl release 104). Transcript flags help to identify the most conserved, high-quality and biologically relevant transcripts as representatives of the studied genes (https://www.ensembl.org/info/genome/genebuild/transcript_quality_tags.html) (Morales et al., 2022). The Study dataset is disclosed in Supplementary Table S1.

### Functional enrichment analysis

g:Profiler web server (https://biit.cs.ut.ee/gprofiler; version e105_eg52_p16_e84549f) (Raudvere et al., 2019) was employed to perform functional enrichment analysis, also known as gene set enrichment analysis (GSEA). The g:GOSt tool performed statistical enrichment analysis to find over-representation of information from Gene Ontology (GO) terms, biological pathways, regulatory DNA elements, human disease gene annotations, and protein-protein interaction networks. g:SCS method was the default method for computing multiple testing correction for *p*-values gained from GO and pathway enrichment analysis. Input data for GSEA analysis can be seen in Supplementary Table S2. This web application was chosen primarily for the reason that it enables a very clear comparison of significantly overrepresented terms between several different lists of genes.

### Apoptosis and DNA repair gene sets

Additional sets of genes defined by the terms cell death/apoptosis (146 genes) and DNA repair (113) were obtained from the Reactome database (https://reactome.org/; version 79) using the Harmonizome tool (Integrated Knowledge About Genes & Proteins; https://maayanlab.cloud/Harmonizome/), in order to independently verify the results of functional enrichment analysis. These two sets of genes are listed in Supplementary Tables S3, S4, including the characteristics and lengths of all exons and introns.

### Human gene expression data

RNA consensus tissue gene data were taken from The Human Protein Atlas database (https://www.proteinatlas.org/; version 21.0). The file downloaded contained consensus transcript expression levels summarized per gene in 55 tissues based on transcriptomics data from HPA (HPA RNA-seq data; https://www.proteinatlas.org/about/assays+annotation#hpa_rna) and GTEx (GTEx RNA-seq data; https://www.proteinatlas.org/about/assays+annotation#gtex_rna). The consensus normalized expression ("nTPM”) value was calculated as the maximum nTPM value for each gene in the two data sources. A set of 422 human genes with nTPM values together with all exon and intron lengths is provided in Supplementary Table S5.

### Statistics

PAST software (PAleontological Statistics, version 4.09, https://www.nhm.uio.no/english/research/resources/past/) was used to evaluate the data using basic statistical methods for comparison of several univariate groups (e.g. the Kruskal-Wallis and Mann-Whitney pairwise tests). The usual threshold for statistical significance (*p* < .05) was accepted. For testing 2 × 2 contingency tables with Fisher’s exact test, which was performed when comparing the Study dataset and the two above-mentioned additional datasets, a freely available calculator from GraphPad (https://www.graphpad.com/quickcalcs/contingency1/) was used.

## Results

### The median number of introns in our human protein-coding gene set is 9

1,427 human genes representing all protein-coding gene groups defined in the HGNC database were included in our study dataset. Approximately 2.3% of genes had no introns. The most frequent numbers of introns in a gene were 4 (6.2%), 1 (6%), 2 (5.8%) and 3 (5.6%) introns. The mean and median number of introns in a gene were 12.6 and 9, respectively (Supplementary Figure S1A). The SYNE1 (spectrin repeat containing nuclear envelope protein 1) gene had the largest number of introns (145). These values of intron numbers do not follow a normal distribution and an excess of genes with fewer introns can be observed (*p* = 2.1E-42, Shapiro-Wilk test, Supplementary Figure S1B).

### 64% of human genes have the longest intron located in the first third of introns, 19% in the second third, and 17% in the third third

**FIGURE 1:**
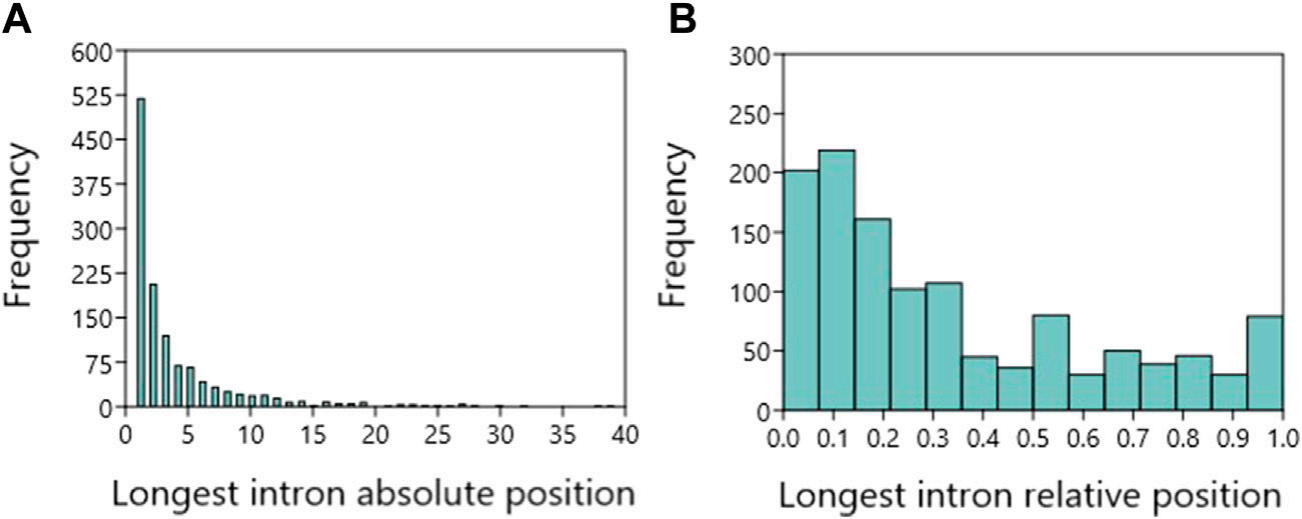
Longest intron positions in human protein coding genes with at least three introns. **(A)**. The frequency of the longest intron absolute positions. **(B)**. The frequency of the longest intron relative positions.

So that the result of the following statistical analysis is not distorted by genes with a small number of introns, we included only genes with three or more introns (*N* = 1,226). The most frequent absolute positions at which the longest intron is found were Intron 1—I1 (42.3%), I2 (16.8), I3 (9.7), I4 (5.6) and I5 (5.4). The longest intron was in absolute position greater than I5 in the remaining 20.1% of genes, with decreasing percentage representation in individual increasing absolute positions (Figure 1A). We defined the relative position as the ratio of the position of the longest intron to the number of all introns in a given gene. Based on the relative positions, the longest intron was located in the first third of introns, i.e. in the interval (0;0.33], in 64% of genes, in the second third in 19.2% and in the third third in 16.8%. The longest intron was in the middle of the gene, i.e. relative position .5, in 5.3% and the longest last intron had 5.9% of genes (Figure 1B).

### The median length of the longest introns decreases with increasing distance from the start of the gene

The median intron length for genes that have only 1 intron (*N* = 85) was 3,085 bp. For genes that have two introns, the median length of the longer intron at position I1 was 4,937 bp (*N* = 48) and at position I2 was 4,691 bp (*N* = 35), the difference not significant.

**FIGURE 2:**
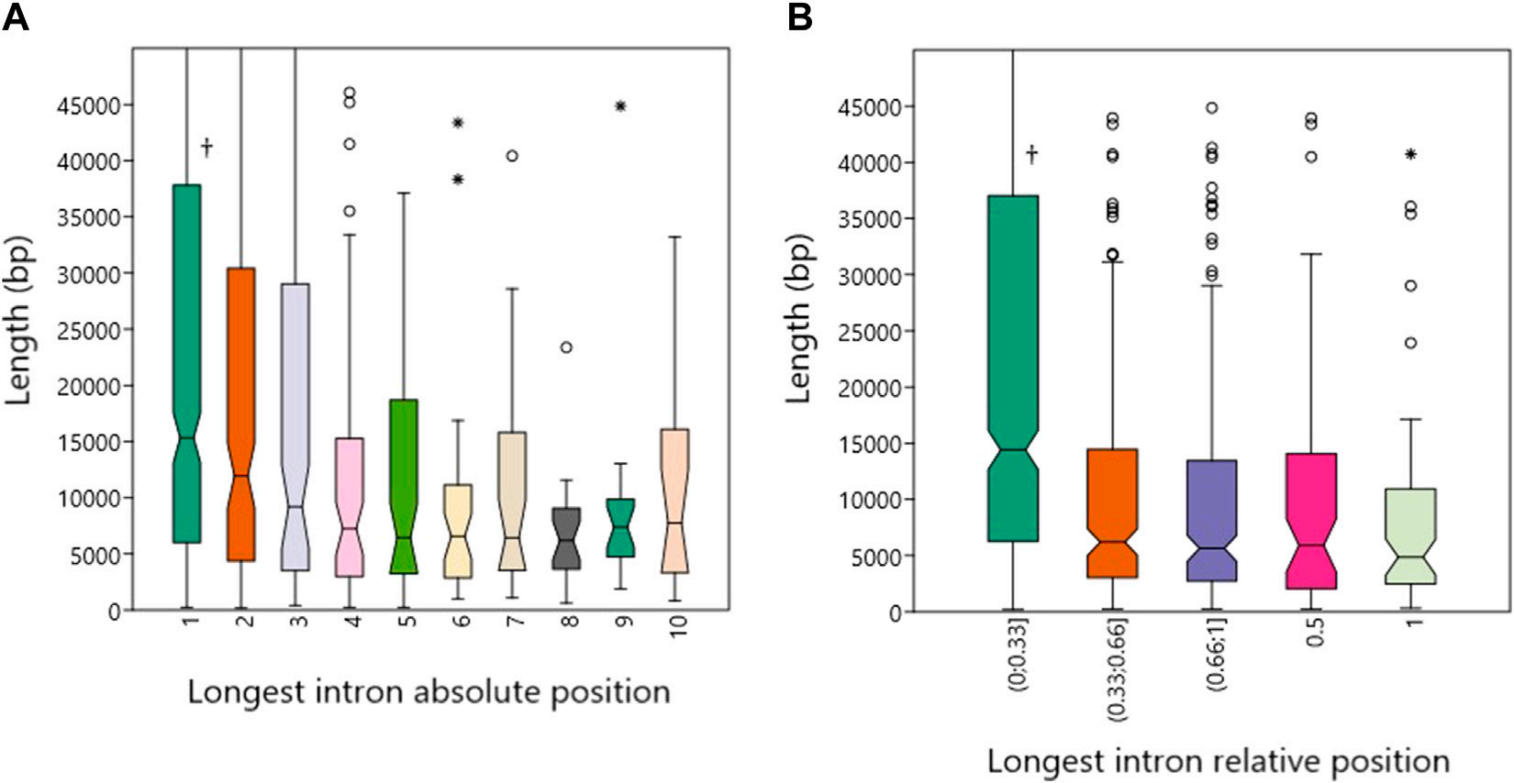
Lengths of the longest introns in human protein-coding genes in relation to their positions. **(A)**. Length dependence on the absolute position of the longest introns. **(B)**. Dependence of length on relative position. Daggers indicate statistically significant differences.

Again, the following part of this analysis only applies to genes with three or more introns. The median lengths of the longest introns located in the first five absolute positions were 15,324, 11,958, 9,192, 7,241 and 6,442 bp (Figure 2A). Only the median in position I1 is significantly higher against the other medians, *p* values are from .02 (vs. I2) to .00007 (vs. I4, Mann-Whitney pairwise test). The median lengths of the longest introns located according to their relative position were 14,404 (in the first third), 6,204 (in the second third), 5,645 (in the third), 5,910 (in the middle) and 4,866 bp (last intron, Figure 2B). Similarly, only the median of the longest introns in the first third of the gene is significantly higher than in the other cases (*p* < .00001).

### The position of the longest intron is related to certain gene biological functions

The results of the GSEA analysis showed that among genes without introns and with one intron there is a significantly higher representation of the biological processes *G protein-coupled receptor signaling pathway* (*p* < .00001) and *sensory perception of chemical stimulus* (*p* < .001). Genes with one intron are additionally enriched with representatives of the biological processes *positive regulation of cytosolic calcium ion concentration*, *nervous system process* and *locomotion* (*p* < .00001 for all). For genes with two introns, we did not detect a specifically higher representation of any biological process.

Genes with three or more introns, which have the longest intron located at the absolute position I1 or I2, interfere significantly in a large number of basic biological processes at the level of the cell as well as the development of a multicellular organism. For genes with the longest intron at I2, statistical significance was specifically increased in the process of *programmed cell death* (*p* < .00001, Table 1). These were, for example, the *BAD* (BCL2 Associated Agonist Of Cell Death), *BAG6* (BAG Cochaperone 6) or *DEDD* (Death Effector Domain Containing) genes. The other tested absolute positions (I3-I9) did not show a specific association with any biological process. Genes with the relative position of the longest intron in the first third showed enrichment for a wide range of biological processes, similar to the absolute positions I1 and I2. The relative position in the second third was significantly associated with the term *reproductive process* (*p* < .001) and in the third with the terms *DNA repair* (*p* < .0001), *mitotic recombination* and *telomere maintenance* (both *p* < .001). Examples of genes associated with the *reproductive process* in our case are *CFTR* (CF Transmembrane Conductance Regulator), *NDC80* (NDC80 Kinetochore Complex Component) and *PANX1* (Pannexin 1). Among the genes included under *DNA repair* were a number of key genes such as *APEX1* (Apurinic/Apyrimidinic Endodeoxyribonuclease 1), *BRCC3* (BRCA1/BRCA2-Containing Complex Subunit 3), *ERCC1* (ERCC Excision Repair 1, Endonuclease Non-Catalytic Subunit), *MLH1* (MutL Homolog 1), *MRE11* (MRE11 Homolog, Double Strand Break Repair Nuclease), *SMC6* (Structural Maintenance Of Chromosomes 6) or *XPA* (XPA, DNA Damage Recognition And Repair Factor).

**TABLE 1:** Significant relationships between the position of the longest intron and gene biological functions based on GSEA.

Absolute position of the longest intron									
GO Term/p-value	Intron 1	Intron 2	Intron 3	Intron 4	Intron 5	Intron 6	Intron 7	Intron 8	Intron 9
cell death	1.8E-04	1.6E-09	ns	ns	ns	ns	ns	ns	ns
programmed cell death	4.0E-04	4.9E-08	ns	ns	ns	ns	ns	ns	ns
apoptotic process	5.0E-04	2.2E-07	ns	ns	ns	ns	ns	ns	ns
regulation of cell death	Ns	2.6E-04	ns	ns	ns	ns	ns	ns	na
regulation of hydrolase activity	Ns	8.1E-04	ns	ns	ns	ns	ns	ns	ns
Relative position of the longest intron									
GO Term/p-value			(0; .33]		(.33; .66]		(.66; 1]		
reproductive process			ns		1.5E-04		ns		
Reproduction			ns		1.8E-04		ns		
DNA duplex unwinding			ns		1.3E-02		ns		
DNA repair			ns		ns		1.4E-05		
DNA metabolic process			ns		ns		5.3E-05		
cellular response to DNA damage stimulus			ns		ns		1.2E-04		
mitotic rekombination			na		ns		2.3E-04		
telomere maintenance			ns		ns		2.9E-04		
double-strand break repair			ns		ns		6.0E-04		
telomere organization			ns		ns		2.0E-03		
regulation of interleukin-1 production			ns		ns		1.7E-02		
interleukin-1 production			ns		ns		1.7E-02		
telomere maintenance via recombination			na		na		1.8E-02		

na, not available; ns, not significant; GO, gene ontology; GSEA, gene set enrichment analysis.

### A higher percentage of DNA repair genes have the longest intron located in the second or third third

The location of the longest introns was monitored for two other gene sets obtained independently of the Study dataset from the Reactome Pathways database. These were Apoptosis (*N* = 146) and DNA repair (*N* = 113) gene sets. While the percentage representation of individual categories of absolute and relative positions in Apoptosis did not differ significantly from the Study dataset, we noted statistically significant deviations in DNA repair. Compared to the Study dataset, among DNA repair genes there is a significantly smaller percentage of genes that have the longest intron at position I1 (absolute position) or in the first third (relative position), *p* < .0001 for both categories. On the contrary, they contain a higher percentage of genes with the longest intron at a position higher than or equal to I5 (*p* < .0001) or in the second and third thirds (*p* < .01 for both; Table 2).

**TABLE 2:** Representation of the absolute and relative positions of the longest introns in the Study dataset and two additional gene sets.

	Study dataset	Apoptosis set^a^	DNA repair set^a^
# genes	1,427	146	113
# genes (≥3 introns)	1,226	126	104
Longest int. abs. position (%)			
I1	42	44	20**
I2	17	16	12
I3	10	11	14
I4	6	10	10
I5	5	2	6
>I5	20	17	38**
Longest int. rel. position (%)			
(0;0.33]	64	62	41**
(.33;0.66]	19	21	30*
(.66;1]	17	17	29*

abs., absolute; int., intron; rel., relative.

^a^ Reactome Pathways Dataset; *, *p* < .01; **, *p* < .0001.

### The highest expression was associated with absolute positions I1 and I2

Analysis mapping the relationship of the absolute and relative position of the longest intron and gene expression was made on a subgroup of 422 genes randomly selected from the Study Dataset. This random selection contained 10 genes without introns (2.4%), 46 genes with one or two introns (10.9%) and 366 genes with an intron number equal or greater than three, which were further tested, as in previous analyzes. The intron data in these genes were linked to expression values determined in 55 human tissues.

The most frequent difference in expression meeting statistical significance was between these groups of absolute positions of the longest intron: I1 higher than I3 (in 12 tissues), I2 higher than I4 (12 tissues), and I2 higher than >I5 (11 tissues; all *p* < .05, Mann-Whitney pairwise test). In general, it can be stated that in most tissues the highest expression was associated with absolute positions I1 and I2. Furthermore, we noted that organ systems could be distinguished based on their own characteristic expression profile (Supplementary Figure S2). A trend towards identical expression in related tissues was also observable when comparing the relative positions of the longest introns (e.g. the highest average expression in the interval (.33;0.66] for CNS tissues; Supplementary Figure S3), however, there was a minimum of relationships meeting statistical significance.

## Discussion

The main interest of our work was to bring more precise and so far unpublished information about the position of the longest introns in human genes and possible connection of the position of the longest intron with biological function or gene expression. Our results confirm the previous information that the longest intron is most often the first intron (Duret, 2001; Bradnam and Korf, 2008; Li et al., 2012), more specifically in 42.3% of human genes having at least three introns. However, as our analyzes show, most human genes do not have the longest first intron. Taking a closer look at not only the absolute but also the relative positions of the longest introns, we see a significant number of genes that have the longest intron in the middle or at the end of the gene, which indicates the possible functional significance of this phenomenon.

Hube and Francastel (2015) reported that in general the median length of introns in human protein-coding genes is 1,520 bp, Piovesan et al. (2019) calculated this value to be 1747 bp. The mention that the first introns tend to be the longest in eukaryotic organisms, or that they are twice as long as other introns, can be found in many works (Duret, 2001; Bradnam and Korf, 2008; Marais et al., 2005; Hong et al., 2006). Li et al. (2012) report that when divided into first introns and non-first introns, the median length in the first group is 3,208 bp and in the second only 1,446 bp in human genes. Not even in recent literature can one find a clearer description of the lengths of the longest introns depending on their localization (Jo and Choi, 2019). The median lengths of the longest introns in our set generally decreased with increasing distance from the start of the gene, from values approximately ten times higher to values four times higher than the above-mentioned general intron median. We also calculated that the median length of the longest introns located at the first absolute position or first third according to relative positons was significantly higher when compared to the other absolute or relative positions. This may illustrate the exceptionality of the longest introns located near the 5′end of genes and possible action by other mechanisms than in introns further away.

It is known that intronless genes, constituting approximately 3% of the human genome, encode mostly for the receptors, signaling and regulatory molecules important in cell growth and proliferation as well as organismal development, with a relatively small proportion of metabolic enzymes (Grzybowska, 2012; Avina-Padilla et al., 2021). G-protein-coupled receptors (GPCRs) and histone-encoding genes form the two largest groups of genes within these genes with a representation of about 50% and 20%, respectively. Furthermore, the expression of intronless genes has been proposed to be highly specialized for neural functions.

No more generally valid information has yet been published on the connection between the length of introns and the biological function of genes. Lopes et al. (2021) concluded recently that smaller genes tend to play a role in functions that are important throughout the whole life. Due to the above-mentioned significantly higher length of introns in the first third, all those with the longest introns in the second and third third can be considered as smaller genes. We have shown in this work that there is a significant relationship between the position of the longest intron and the function of some genes. We demonstrated this on the example of a clinically important group of DNA repair genes, which have the longest intron more often in the second or third third.

The influence of introns on the expression of host genes has been described as IME and is a different mechanism than the action of long-known elements such as promoters, silencers or enhancers. Rose et al., 2011, showed in experiments with plant introns that IME acts at the level of DNA rather than nascent RNA and probably depends on the physical properties of introns than factors that bind to it. Information about average transcription elongation rates and role of histone post-translational modifications indicates that gene architecture contributes to the establishment of gene-specific transcription elongation rates that vary within an order of magnitude (Heyn et al., 2014). In this sense, the presence and abundance of intron sequences is essential. Experimentally, the positive influence of introns near the beginning of transcription and especially in the 5′untranslated regions of genes was shown (Shaul, 2017; Crane et al., 2019).

Our sample of 1,427 human genes representing all groups of protein-coding genes defined in the HGNC database showed higher mean and median values (12.6 and 9) of the number of introns in genes than already published works that worked with whole-genome data. Gorlova et al. (2014) described the average number of introns per human gene as 8–9. Piovesan et al. (2019) calculated the median number of introns in human protein-coding genes to be eight and characterized this figure as long-term stable in contrast to, for example, the number of all these genes in the human genome. From this view, the main limiting factor of this study is the relatively small size of the tested set of genes and we anticipate that the results of this pilot study will be refined by subsequent studies that will consider all genes in the human or other genome.

The influence of gene expression by the location of the longest intron has not yet been discussed in the literature. Our results show that such a relationship is very likely to exist and is most evident for absolute positions I1 and I2. Most likely, the total number of exons and introns and their lengths play a more fundamental role in the rate of transcription and, consequently, in overall expression. Our results are consistent with the assertion of Shaul (2017), where he writes that there is no unequivocal answer to the question of the way introns influence gene expression, and that it is a specific mix of processes in each individual case. However, the data presented in this article indicate that the position of the longest intron is another of the subtle mechanisms tuning gene expression in human cells and tissues.

## Data Availability

The original contributions presented in the study are included in the article/Supplementary Material, further inquiries can be directed to the corresponding author.

## Supplementary material

SUPPLEMENTARY FIGURE S1 Number of introns in human protein-coding genes—whole dataset of 1427 genes. A. The frequency of the number of introns. B. The normal probability plot for the number of all introns (correlation coefficient is 0.86).

SUPPLEMENTARY FIGURE S2 Gene expression divided by the absolute position of the longest intron in human tissues produces characteristic profiles. **(A)** Mean and whisker plots showing the CNS Profile on 3 selected tissues. **(B)** Plots representing the Lymphatic System Profile.

SUPPLEMENTARY FIGURE S3 Gene expression sorted according to the relative position of the longest intron in human protein-coding genes—characteristic tissue profiles. **(A)** Mean and whisker plots showing the CNS Profile on 3 selected tissues. **(B)** Plots representing the Lymphatic System Profile.
