# Supplementary figures and images for: The position of the longest intron is related to biological functions in some human genes
Source: Front Genet. 2023 Jan 10;13:1085139. doi: 10.3389/fgene.2022.1085139 (PMC9875286; doi:10.3389/fgene.2022.1085139)

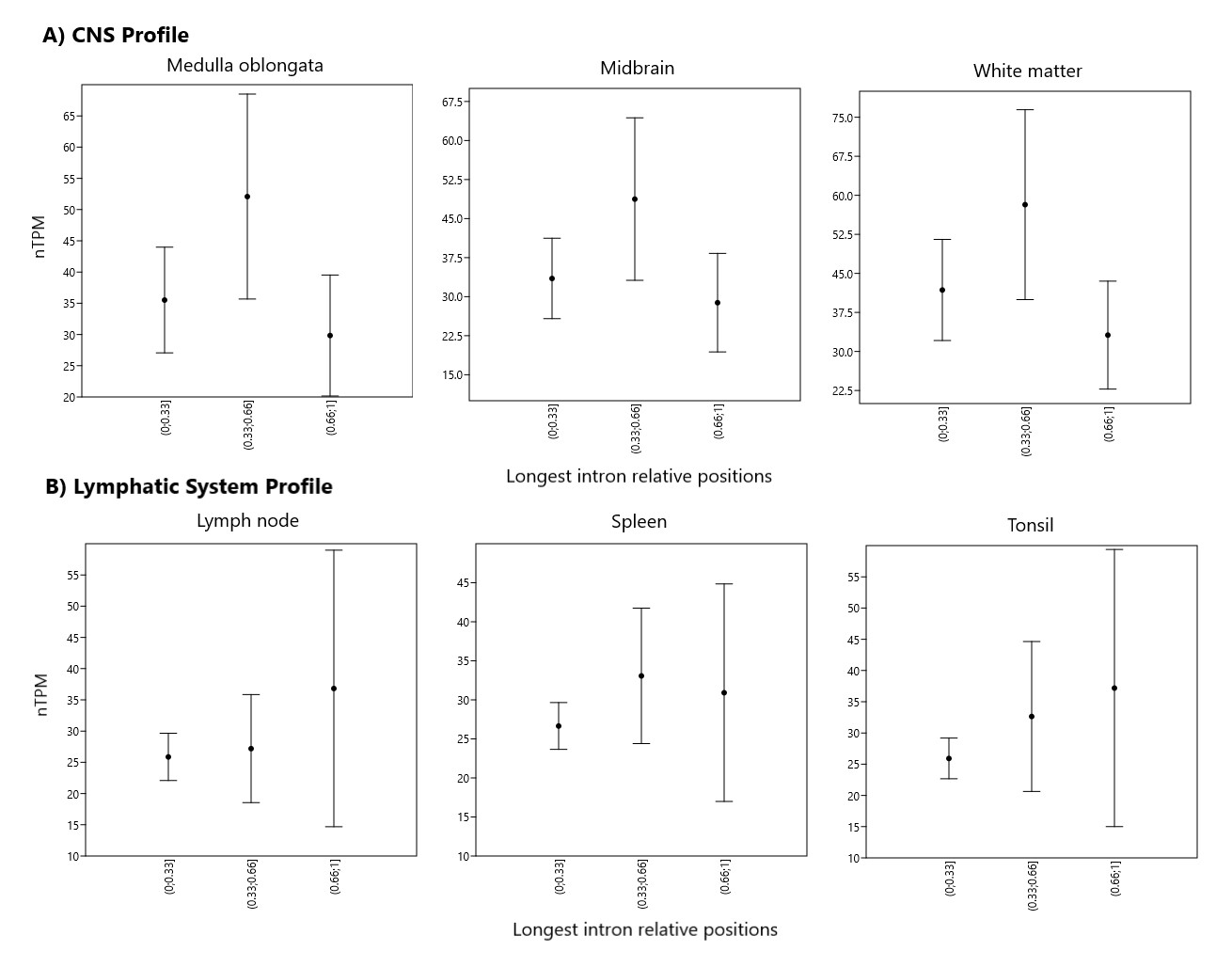

Supplement: Supplementary file 3 [file Image3.jpg]

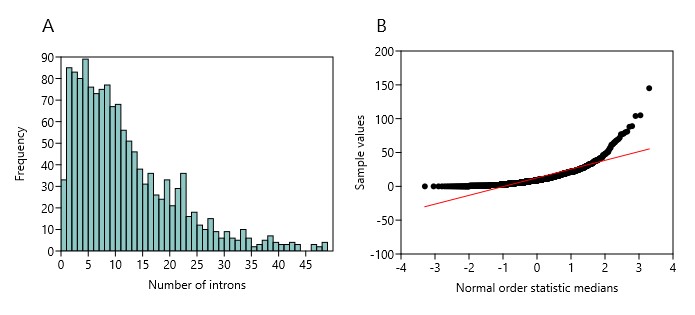

Supplement: Supplementary file 4 [file Image1.JPEG]

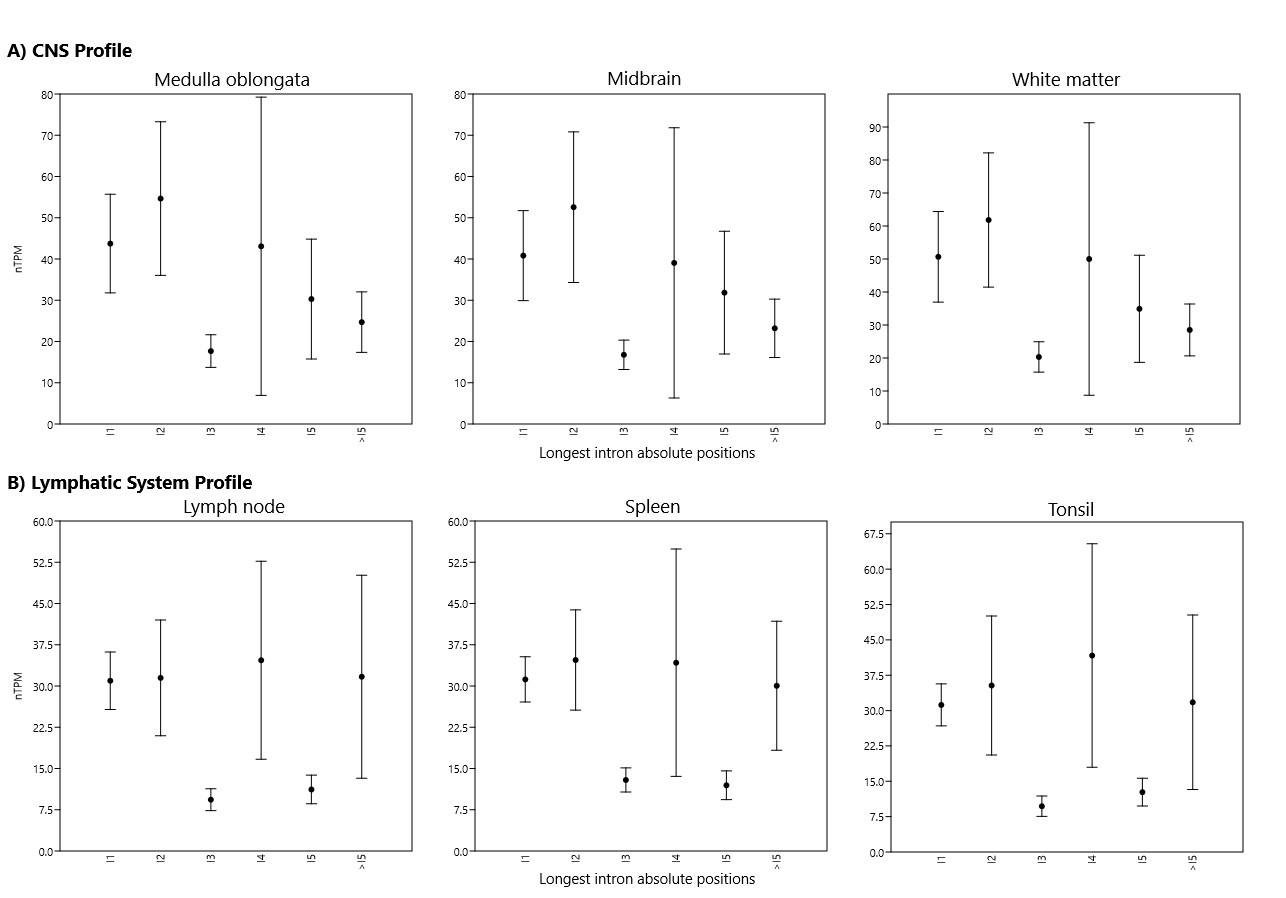

Supplement: Supplementary file 5 [file Image2.JPEG]
